# Ciprofloxacin Treatment in Juvenile Mice Involves Neuronal Activation and Mimics Physical Features of Human Disease

**DOI:** 10.1002/jor.70095

**Published:** 2025-10-28

**Authors:** Nicole A. Chittim, Amro A. Hussien, Nicolo Dubacher, Gabor Matyas, Jess G. Snedeker

**Affiliations:** ^1^ Department of Orthopedics Balgrist University Hospital Zurich, University of Zurich Switzerland; ^2^ Institute for Biomechanics ETH Zurich Zurich Switzerland; ^3^ Department of Cell and Tissue Dynamics Max Planck Institute for Molecular Biomedicine Münster Germany; ^4^ Center for Cardiovascular Genetics and Gene Diagnostics Foundation for People with Rare Diseases Schlieren Switzerland; ^5^ Zurich Center for Integrative Human Physiology University of Zurich Zurich Switzerland

**Keywords:** ciprofloxacin, fluoroquinolone, tendinopathy, tendon

## Abstract

Tendinopathy is a complex, painful condition that affects up to 5% of the general population in their lifetime. Antibiotic treatment with fluoroquinolones has been associated with the onset of tendinopathy and tendon rupture. The mechanisms behind fluoroquinolone induced tendinopathy remain unclear. To probe activation of potentially causative pathways, we treated juvenile mice with ciprofloxacin in drinking water for 4 weeks and performed RNA sequencing on tail tendons. We discovered that ciprofloxacin‐treated mice had upregulated genes relating to nerve development. Additionally, treated mice showed downregulation of genes associated with extracellular matrix (ECM) processes. We further explored ECM changes using histological and mechanical testing methods on patellar tendons. We found that ciprofloxacin treatment led to altered cell morphology and proteoglycan density. These changes translated to a decrease in mechanical properties of the patellar tendons. Furthermore, ciprofloxacin‐treated mice had a higher percentage of apoptotic cells, and we confirmed increased presence of nerve cells (Plexin B1^+^) in the patellar tendons compared to controls. Taken together, we showed that ciprofloxacin treatment in juvenile mice induces structural and biological phenotypes commonly associated with fluoroquinolone‐induced tendinopathy and identify the axis of pathological neural activation as a promising area for further exploration.

**Clinical significance:** Oral administration of ciprofloxacin in mice presents a clinically relevant model for studying mechanisms of tendinopathy in humans.

## 1 Introduction

Tendons have a poor regenerative ability, and injuries can cause long‐term pain for the patient with few effective treatments [1]. Tendinopathy describes biologically driven tendon disorders, but generally features the following characteristics: disorganized collagen fibers, disrupted ECM including proteoglycan accumulation, cellular apoptosis, decreased mechanical properties, and increased immune and inflammation markers, angiogenesis, and innervation [2, 3, 4].

Fluoroquinolones are a class of broad‐spectrum antibiotics that have been associated with tendinopathy and tendon rupture in humans [5, 6]. Ciprofloxacin has been reported as the most commonly prescribed second‐generation fluoroquinolone, and is commonly prescribed for high‐level athletes [7, 8, 9]. It is an effective antibiotic for a broad spectrum of bacteria, particularly those resistant to other antibacterial agents and exhibits good pharmacokinetic properties with minimal toxicities [5, 6]. Most adverse effects experienced from ciprofloxacin therapy are clinically trivial, affecting the gastrointestinal, neurological or immune systems [9].

Ciprofloxacin‐induced tendon symptoms are reported to occur in patients approximately 2 weeks after starting treatment, with tendon ruptures commonly occurring in such cases within 1 month of starting treatment [9]. The risk of experiencing tendinopathic symptoms following fluoroquinolone use is reported to decrease over time after stopping fluoroquinolone therapy [10]. In a study of 10 patients using fluoroquinolone drugs, 6 of 10 developed tendonitis and 4 of 10 experienced tendon rupture [5]. Most studies report injuries to the Achilles tendon, with injuries also reported in the patellar tendon (PT), quadriceps tendon, and rotator cuff [11]. Other factors, such as age and systemic diseases like diabetes, can increase the risk of fluoroquinolone‐induced tendon rupture [10]. Due to its adverse effects on the musculoskeletal system, fluoroquinolone use in adolescents is limited [8]. An 18‐year study enrolling millions of adolescents reported a 90‐day tendon rupture rate of 0.0136% with fluoroquinolone use versus 0.0116% for comparable antibiotics, concluding no increased risk of tendon rupture associated with fluoroquinolone use for adolescents [8], although potential effects on tendon development and long‐term health have not been investigated and reported.

Many studies have investigated potential mechanisms of action of ciprofloxacin on tendons. *In vitro* studies of tendon fibroblasts cultured with ciprofloxacin have discovered that the drug decreases fibroblast and tenocyte proliferation [12]; induces apoptosis [13]; decreases collagen synthesis [12], specifically collagen type I [13, 14]; and increases matrix‐degrading proteases [13, 14], thus decreasing the mechanical integrity of the tendon [12]. *Ex vivo* tendon explant studies have reported similar findings: ciprofloxacin treatment of explants led to decreased cell viability [15], cell migration [14], inhibition of proteoglycan degradation [15], increased degradation of type 1 collagen [16], and increased MMPs [16]. However, the mechanisms of fluoroquinolone‐induced tendinopathy are still unclear.

Here, we explored the tendinopathic phenotypes that emerge from ciprofloxacin treatment in juvenile mice and screen for activation of cellular signaling pathways. We examined available tendons of mice administered orally with ciprofloxacin via drinking water for 4 weeks. We discovered that ciprofloxacin‐treated mice developed relevant structural and biological phenotypes in their tail tendon fascicles (TTFs) and PTs reminiscent of human tendon disease, and that these changes are associated with neural activation within tendon tissues.

## 2 Methods

### 2.1 Study Approval

Institutional and local guidelines were followed for animal experimentation and maintenance (PE/EA/407‐7/2020, Hungary), as well as the EU Directive 2010/63/EU for animal experiments.

### 2.2 Ciprofloxacin Treatment in Mice

C57BL/6 mice (Charles River Laboratories, Wilmington, MA, USA) of both sexes were bred and housed with a 12‐h light/dark cycle. They received standard rodent chow and were allowed water ad libitum. At 4 weeks of age, mice began oral treatment of approximately 750 mg/kg bw per day ciprofloxacin (Ciprofloxacin 1 A Pharma) in drinking water, based on reported water consumption of mice when available ad libitum (*n* = 10) [17]. Age‐ and genotype‐matched untreated mice (*n* = 20) served as controls, receiving drinking water without ciprofloxacin. Water consumption was monitored to confirm all mice drank similar amounts. Animal weights were recorded at the beginning and end of treatment. After 4 weeks of treatment, mice were euthanized and immediately frozen at −20°C.

### 2.3 RNA Extraction and Sequencing

Frozen tendons were thawed, isolated and snap frozen in liquid nitrogen. Tissue was milled and homogenized in Genezol. Samples were centrifuged with chloroform, and the supernatant was used with the RNeasy Micro Kit (Qiagen, 74004) following manufacturer's instructions. RNA concentration was measured using a Qubit 4.0 Fluorometer (Invitrogen, Carlsbad, CA), and TTF samples were sent to GENEWIZ (Leipzip, Germany) for RNA sequencing library preparation. More information on sequencing and analysis can be found in Supporting Information. PT samples were used for targeted RT‐qPCR to confirm RNA sequencing results.

### 2.4 Tissue Sectioning and Histological Analysis

The PT was cut in 10 µm sections. Sections were stained with hematoxylin and eosin (H&E, Merk, 115938), picrosirius red (PSR, Polysciences Inc., 24901‐250) and alcian blue. Sections for histological staining were chosen at random with no preference towards male/female or left/right legs, and all analysis was performed in a blinded manner with FIJI (ImageJ, v2.3.0/1.53q). H&E sections were evaluated for cell morphology, defining elongated cells as being longer than wide in the direction of fibers. PSR sections under light microscopy (Supporting Figure 3) were analyzed for percentage of collagen in each section and fiber crimp, defined as the shortest straight end‐to‐end distance of an individual fiber divided by the end‐to‐end length of the fiber following the curves [18]. PSR sections under polarized light microscopy were quantified for the distribution of birefringence in the tendon mid‐substance region using previously defined hue thresholds for red (2–9, 230–256), orange [10, 11, 12, 13, 14, 15, 16, 17, 18, 19, 20, 21, 22, 23, 24, 25, 26, 27, 28, 29, 30, 31, 32, 33, 34, 35, 36, 37, 38], yellow (39–51), and green (52–128) [19]. Alcian blue sections were quantified for the percentage of proteoglycans in a section.

### 2.5 TUNEL Assay

A TUNEL assay for apoptosis (Abcam, ab206386) was performed following manufacturer's instructions for tissue cryosections, including a positive and negative control (Supporting Figure 5). Stained sections were analyzed following manufacturer's definition of positive staining for apoptosis. Values are reported as percentages of apoptotic cells of the entire cell population in the frame.

### 2.6 Immunohistochemistry

Frozen sections were dried at room temperature (RT), fixed with 4% PFA and washed with PBS. Sections were blocked with 3% BSA for 1 h and incubated at 4°C overnight with primary antibodies. Samples were washed with PBS and incubated for 1 h with secondary antibodies. Sections were washed with PBS, incubated with NucBlue (Thermo Fisher Scientific R37606), washed again with PBS and MilliQ water, mounted and imaged (Nikon Eclipse Ti2 inverted with lasers at 405 nm, 561 nm and 640 nm).

### 2.7 DMMB Assay

Frozen PTs were digested in lysis buffer and proteinase K based on previously defined protocols [20]. DNA concentration was determined using a microplate spectrophotometer (Biotek Epoch, CA USA). DMMB solution was prepared as previously described [20]. Chondroitin‐sulfate A (Sigma‐Aldrich, C‐9819) was used for a standard curve [20]. Absorbance for each sample was read in triplicate at 535 nm, and the concentration of sGAGs was extrapolated using the standard curve. The concentration of sGAGs for each sample was normalized to DNA concentration and reported as a ratio of sGAGs:DNA.

### 2.8 Patellar Tendon Ramp‐To‐Failure Testing and Analysis

The jig was fabricated and designed to fit in a universal testing machine (Zwick Z010 TN). Information on jig sensitivity testing can be found in Supporting Information. Tensile testing of the PT was performed blinded. Two hind legs were received on ice and stored at −20°C. On the day of testing, hind legs were thawed, and PTs were isolated with attached tibia and patella into 1% PBS. The width and thickness of the tendon was measured using an inverted microscope (Motic AE2000, 4x). The unit was inserted into the jig and kept in 1% PBS for test duration. The tendon was stretched to a preload of 0.5 N, preconditioned 25 cycles to 1% strain, allowed a 30 s rest period and stretched to failure at 5.0% L_0_/s [21]. Force and position data were recorded. Cross‐sectional area (CSA) was used to calculate stress. Elastic modulus was calculated as the linear region of the stress‐strain curves, failure stress as maximum stress and failure strain as the corresponding strain value [22].

### 2.9 Statistics

Statistical analysis was performed using GraphPad Prism 9.2.0 (GraphPad Software, CA, USA). Statistical analysis of RNA sequencing data was taken as the adjusted p‐value from differential expression via the SUSHI framework of the Functional Genomics Center Zurich (ETH Zurich and the University of Zurich) [23]. Analysis of histological data was performed using a nonparametric Mann‐Whitney test and Cohen's *d* effect size. Mechanical data was analyzed with a two‐sided independent *t*‐test and Cohen's *d* effect size. Similar findings were reported for both sexes.

## 3 Results

### 3.1 Systemic Administration of Ciprofloxacin Alters Tendon Transcriptome

**Figure 1:**
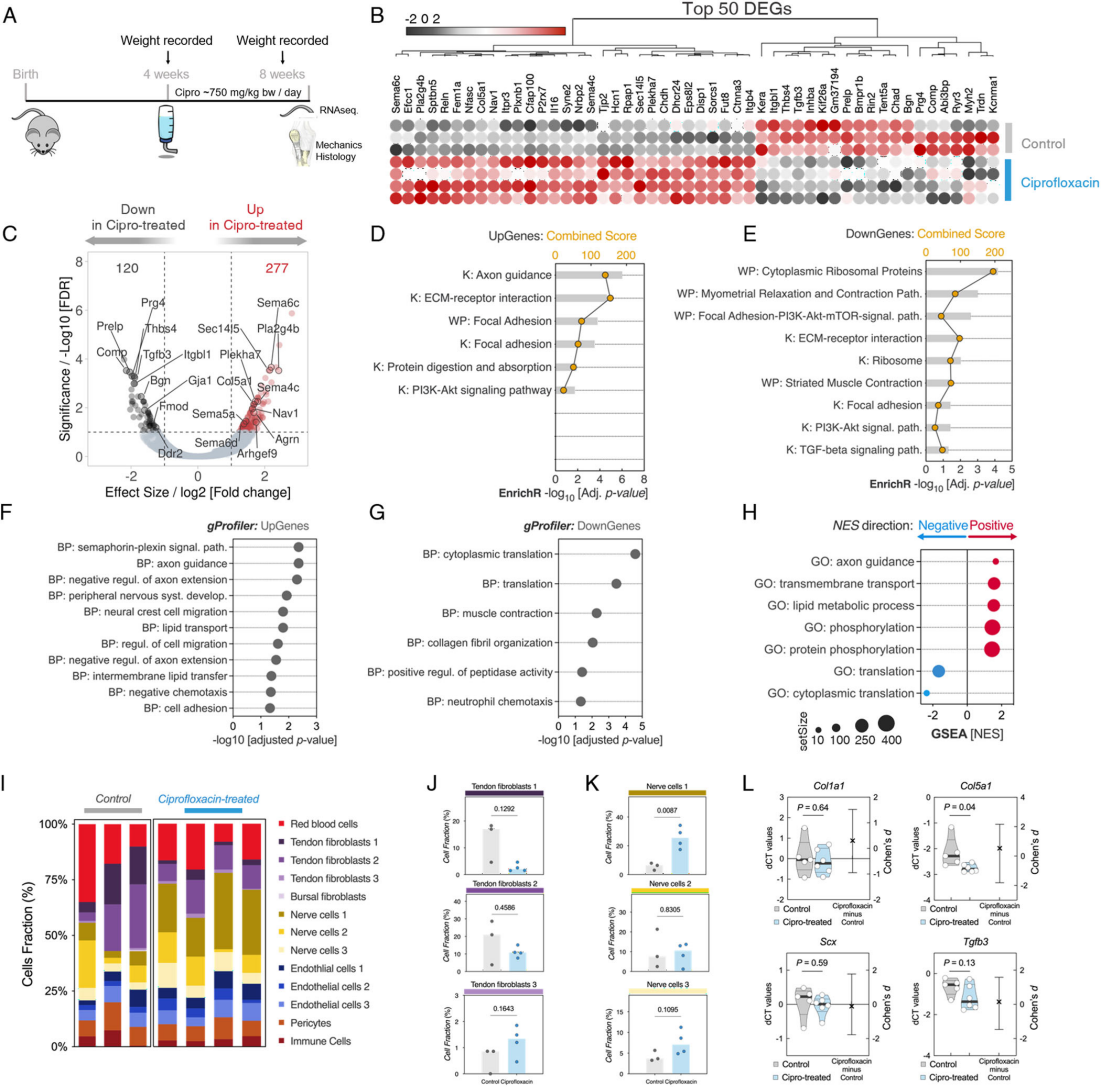
RNA sequencing analysis of tendons from ciprofloxacin‐treated mice. (A) Schematic representation of the timeline of ciprofloxacin treatment of mice. (B) Heatmap of the top 50 differentially expressed genes (DEG) in ciprofloxacin‐treated versus untreated control. Rows depict individual samples (*N* = 4 mice for ciprofloxacin‐treated, *N* = 3 untreated). Red denotes upregulated genes, black denotes downregulated genes. (C) Volcano plot of the RNA‐seq DEGs of ciprofloxacin‐treated versus untreated controls. Colored dots indicate the 397 significantly expressed genes, as determined by EdgR algorithm, with the horizontal line corresponding to a *p* ≤ 0.01 and vertical lines are log_2_[Fold‐change] ± 1. (D, E) Enrichr pathways analysis of upregulated (D) and downregulated (E) DEGs, queried against WikiPathways (WP), and KEGG (K) databases. Only results with an adjusted *p*‐value < 0.05 are shown. (F, G) Overrepresentation analysis (ORA) of Biological Processes (BP) GO terms of (F) upregulated and (G) downregulated DEGs. Statistical cutoff was set using a hypergeometric over‐representation test with significance at *q*‐value < 0.05. (H) Pre‐ranked Gene Set Enrichment Analysis (GSEA) of positively and negatively enriched GO terms in ciprofloxacin‐treated tendons. NES: Normalized Enrichment Score. (I–K) CibersortX deconvolution analysis of inferred cellular composition in ciprofloxacin‐treated and untreated controls. (L) Targeted RT‐qPCR of the patellar tendon. Primers chosen based on tail tendon whole transcriptomic data analysis. Each point represents an average dCT value from two replicates from one animal (*n* = 5 control, *n* = 6 ciprofloxacin‐treated mice). *p*‐values and Cohen's *d* estimated effect size are displayed.

To broadly explore the influence of ciprofloxacin on tendon transcriptome, TTFs from mice treated orally with ciprofloxacin for 4 weeks were collected for RNA‐sequencing (Figure 1A). Using a *p* value of ≤ 0.01 and a log_2_ fold‐change of ±1, we found that ciprofloxacin administration altered the mRNA expression levels of 397 genes compared to untreated controls (Figure 1B,C). We identified 120 downregulated differentially expressed genes (DEGs) in the ciprofloxacin‐treated group, many of which are typically expressed in tendon ECM, including *Bgn, Fmod, Tgfb3*, and *Comp*. We also identified 277 upregulated DEGs in the ciprofloxacin‐treated group including those involved in inflammation, like *Il16*, and in neuronal ingrowth, like *Sema6c, Sema4c*, and *Plxnb1*. Using *Enrichr*, we identified pathways related to axonal guidance that were upregulated in the ciprofloxacin‐treated group (Figure 1D). Downregulated genes were enriched in pathways associated with ribosomal processes, muscle contraction, and TGF‐beta signaling (Figure 1E). Pathways suggestive of PI3K‐Akt signaling, ECM‐receptor interaction and focal adhesion processes were enriched in both upregulated and downregulated genes (Figure 1D,E). Overrepresentation analysis (ORA) of the Gene Ontology biological processes (GO‐BP) and Gene Set Enrichment analyses further confirmed the enrichment of these transcriptional processes (Figure 1F–H). We found many processes relating to nervous system development, axon guidance, and cell migration were upregulated in the ciprofloxacin‐treated group (Figure 1F). Specifically, many genes in the semaphorin family were upregulated in the ciprofloxacin‐treated group contributing to upregulated BP terms like semaphorin‐plexin signaling pathway and peripheral nervous system development. In contrast, biological processes relating to translation and collagen fibril organization were downregulated in the ciprofloxacin‐treated group when compared to the control group (Figure 1G,H).

To link these findings to a subpopulation of cells in TTFs, we performed CIBERSORTx digital cytometry to predict cell populations within the tendon. Using previously published scRNA‐seq datasets [24], we generated cell type‐specific “signature matrix” of marker genes enriched in the most abundant cell types in tendon, including fibroblast subpopulations, endothelial cells, pericytes, immune cells, and nerve cells. Enumeration of cell subpopulation revealed similar frequencies of endothelial cells, pericytes, and immune cells in both groups; however, ciprofloxacin‐treated tendons are predicted to have smaller portions of tendon fibroblasts compared to untreated controls (Figure 1I,J). The ciprofloxacin‐treated group also had a significantly higher predicted fraction of nerve cells in their tendons than the control group (*p* = 0.0057) (Figure 1K).

Targeted RT‐qPCR was performed using the PTs of mice to verify the translatability of TTF RNA sequencing results to the PT. *Col5a1* was identified as a top 50 upregulated DEG in the TTF of the ciprofloxacin‐treated group and was also confirmed as having significantly lower dCT values in the PT of the ciprofloxacin‐treated group, indicating upregulation (Figure 1L, *p* = 0.04). Although *Tgfb3* was identified as a top 50 downregulated DEG in TTF of the ciprofloxacin‐treated, we did not find a significant difference in the PT (*p* = 0.13). We also did not identify any significant differences in *Col1a1* or *Scx* in the PT (*p* = 0.64 and 0.59, respectively).

Collectively, these analyses suggest that systemic administration of ciprofloxacin significantly alters the tendon transcriptome in mouse TTF, with the enrichment of several transcriptional processes predictive of cell‐ECM interactions and neuronal processes.

### 3.2 Ciprofloxacin Treatment Alters Tendon ECM in Mice

**Figure 2:**
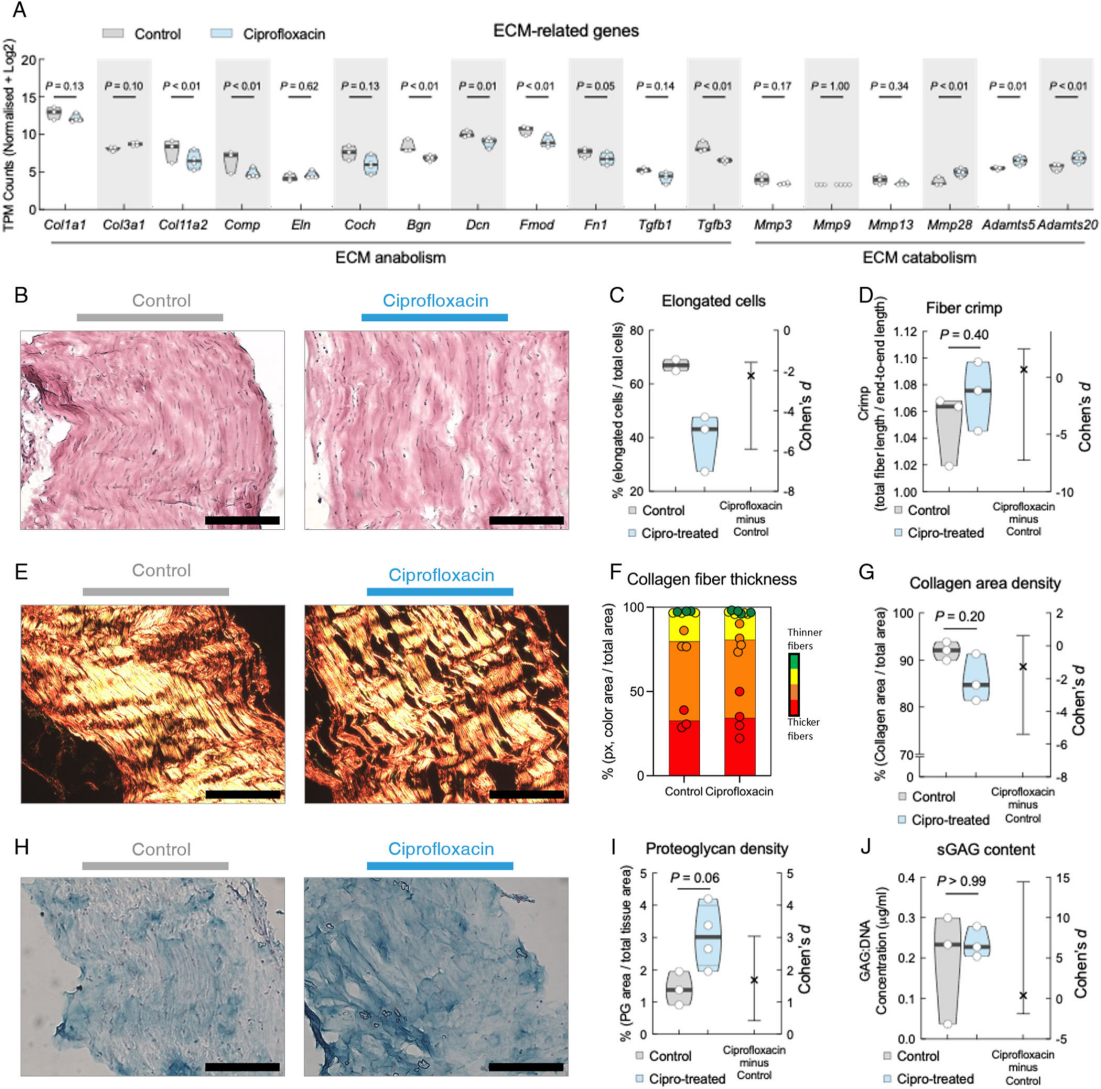
Systemic ciprofloxacin treatment results in altered ECM transcriptome in tail tendons and histological changes in patellar tendon ECM. (A) Normalized transcripts of ECM‐related genes from RNA sequencing data. Sorted by anabolic (fibrillar and proteoglycan) and catabolic genes. (B) Representative images of H&E staining of control (left) and ciprofloxacin‐treated (right) patellar tendon midsubstance region. (C) Quantification of cell morphology as calculated by percentage of elongated cells to total cells. (D) Analysis of collagen fiber crimp as calculated by total fiber length following the curves of the fiber divided by end‐to‐end length. (E) Representative images of Picrosirius red (PSR) staining under polarized light of control (left) and ciprofloxacin‐treated (right) patellar tendon midsubstance region. (F) Quantification of collagen fiber thickness, calculated by separating the colors from PSR images, with red being thickest fiber and green being thinnest. Bars indicate average ± standard deviation. (G) Quantification of collagen area density. (H) Representative images of Alcian blue staining of control (left) and ciprofloxacin‐treated (right) patellar tendon midsubstance region. (I) Quantification of proteoglycan density from Alcian blue images. (J) Sulphated GAG content quantified from DMMB assay. Scale bars are 200 μm. Violin plots show the distribution of the data with the median, first and third quartiles highlighted. Each dot represents an average from 5 to 10 histological sections, *n* = 2–4 mice. Statistical analysis done with unpaired *t*‐test. *p*‐values and Cohen's *d* estimated effect size are displayed.

Given the enrichment of ECM signature in the transcriptome analysis and the tendinopathic alteration to ECM [25, 26], we examined key genes related to tendon ECM. Expression levels of many genes related to tendon ECM anabolism, including biglycan (*Bgn*), fibromodulin (*Fmod*), and cartilage oligomeric matrix protein (*Comp*) were downregulated in TTFs of ciprofloxacin‐treated mice. In contrast, transcripts of catabolic ECM genes such as a disintegrin and metalloproteinase with thrombospondin motifs (ADAMTS) and matrix metalloproteinases (MMPs), were upregulated in TTFs of ciprofloxacin‐treated mice (Figure 2A).

We then performed histology on the PT to confirm similar ECM changes are occurring in load‐bearing tendons as those found in the transcriptome of the TTFs. PTs from both groups were stained with H&E or PSR (Figure 2B and E, respectively). Quantitative analyses of cell morphology revealed PTs from ciprofloxacin‐treated mice had less elongated tendon cells when compared to control mice with a large effect size (Cohen's *d* = −2.25, 95% CI [−5.91, −1.59]) (Figure 2C). We also found that the PTs from ciprofloxacin‐treated mice showed a trend towards having a higher crimp ratio (*p* = 0.40) with a moderate effect size (Cohen's *d* = 0.68, 95% CI [−7.25, 2.48]) (Figure 2D). Analysis of collagen density from PSR staining showed no statistical significance between the groups (*p* = 0.20), but high effect size suggesting meaningful difference (Cohen's *d* = −1.28, 95% CI [−5.39, 0.63]) (Figure 2G). Collagen density was further analyzed under polarized light, since polarization refracts light differently based on collagen fiber thickness [19]. No differences in fiber thickness were observed (Figure 2F).

Next, we examined proteoglycans and sulfated glycosaminoglycans (sGAGs) using Alcian blue and DMMB assays, respectively, in PTs. We found that the density of proteoglycans in the tendon midsubstance of the ciprofloxacin‐treated group was higher than the control group (*p* = 0.06, Cohen's *d* = 1.68, 95% CI [0.43, 3.04], Figure 2I). The DMMB assay results varied widely across specimens and revealed no measurable difference in sGAG concentration after normalizing for cellularity (*p* > 0.99, Cohen's *d* = 0.37, 95% CI [−1.87, 14.46], Figure 2J).

Taken together, these analyses suggest that ciprofloxacin treatment alters the ECM in both TTFs and PTs, including changing the morphology of tendon cells with visual evidence of increased tendon tissue proteoglycan content.

### 3.3 Ciprofloxacin Treatment Affects PT Mechanics in Mice

**Figure 3:**
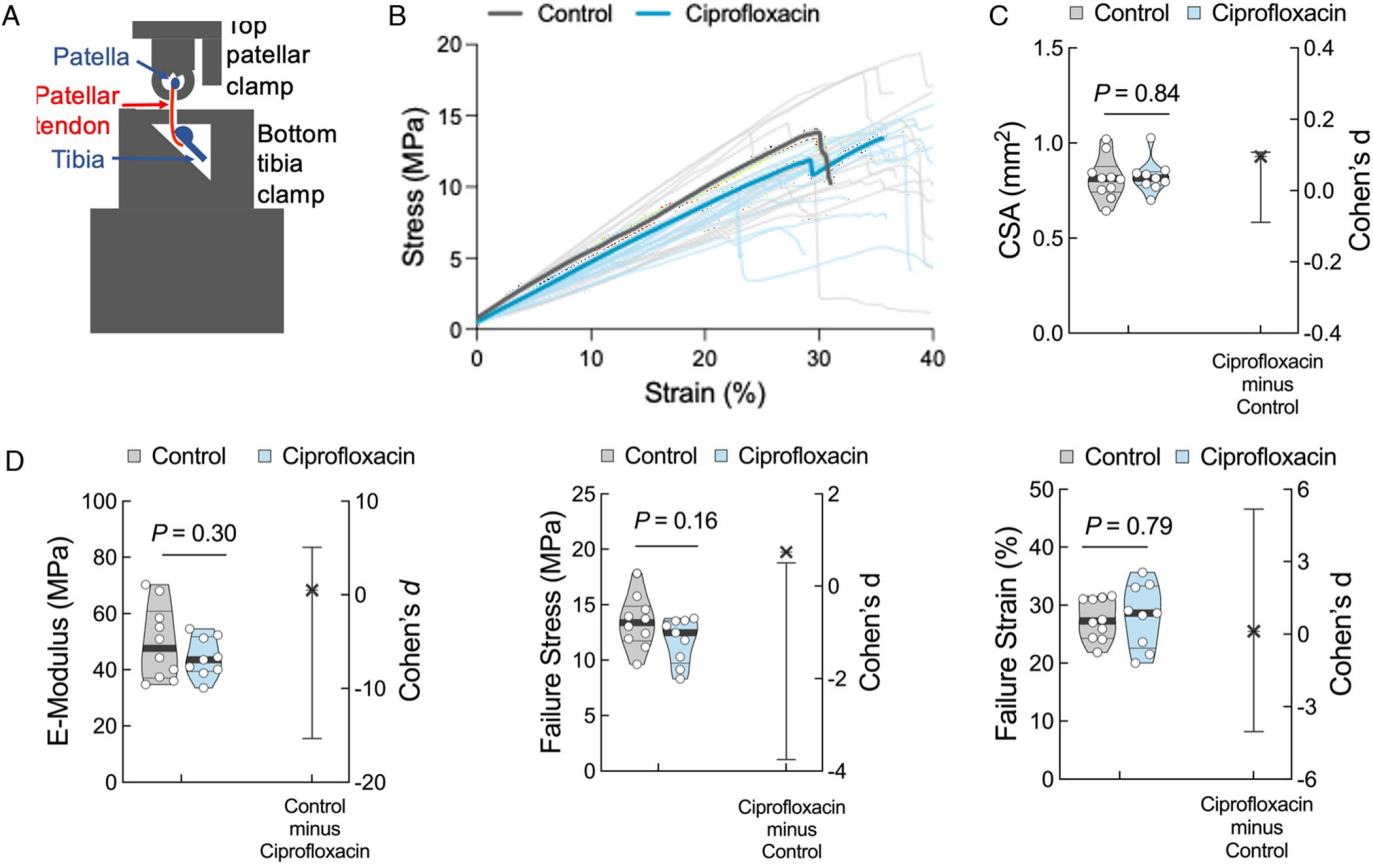
Patellar tendon mechanics of ciprofloxacin‐treated mice show a moderate to large effect size compared to untreated controls. (A) Schematic of the custom‐fabricated jig, which fits into a universal testing machine Zwick. (B) Ramp‐to‐failure curves of the patellar tendon of the control (gray, average curve bold, *n* = 18 tendons in 10 mice) and ciprofloxacin‐treated (blue, average curve bold, *n* = 18 tendons in 9 mice) groups. (C) Cross‐sectional area and (D) mechanical properties with *p*‐values and Cohen's *d* effect size. Violin plots show the distribution of the data with the median, first and third quartiles highlighted. Each point represents one animal, i.e., the average of the left and right patellar tendons from a single animal, *n* = 10 for the control group and *n* = 9 for the ciprofloxacin group. The difference axis of the estimation plot displays the Cohen's *d* effect size with its 95% confidence interval illustrated by the vertical black line. *p*‐value is calculated with a two‐sided independent *t*‐test.

We designed and fabricated a custom jig for the mechanical testing of the murine PT (Figure 3A). We measured the mechanical properties of the load‐bearing PT since it is commonly affected by ciprofloxacin use, yet seldomly studied [7, 12]. Initial calibrations proved that our setup is sensitive, reproducible, and yielded similar mechanical properties to those reported elsewhere for murine patellar tendons (Supporting Figure 4) [21, 27].

Mice hind legs were thawed, PTs were isolated and CSA was measured (Figure 3C), and the tendons were tensile tested until rupture while force and position values were recorded to later calculate mechanical properties (Figure 3B,D). Treatment with ciprofloxacin did not affect the CSA, suggesting that the drug caused neither atrophy nor hypertrophy of the tendon. Although no statistically significant differences were identified, effect sizes for elastic modulus and failure stress suggest possible group differences (Cohen's *d* = 0.49, 95% CI [−15.35, 5.07] and 0.74, 95% CI [−3.75, 0.50], respectively), although being underpowered.

### 3.4 Ciprofloxacin Treatment Induces Biological Changes in PT

**Figure 4:**
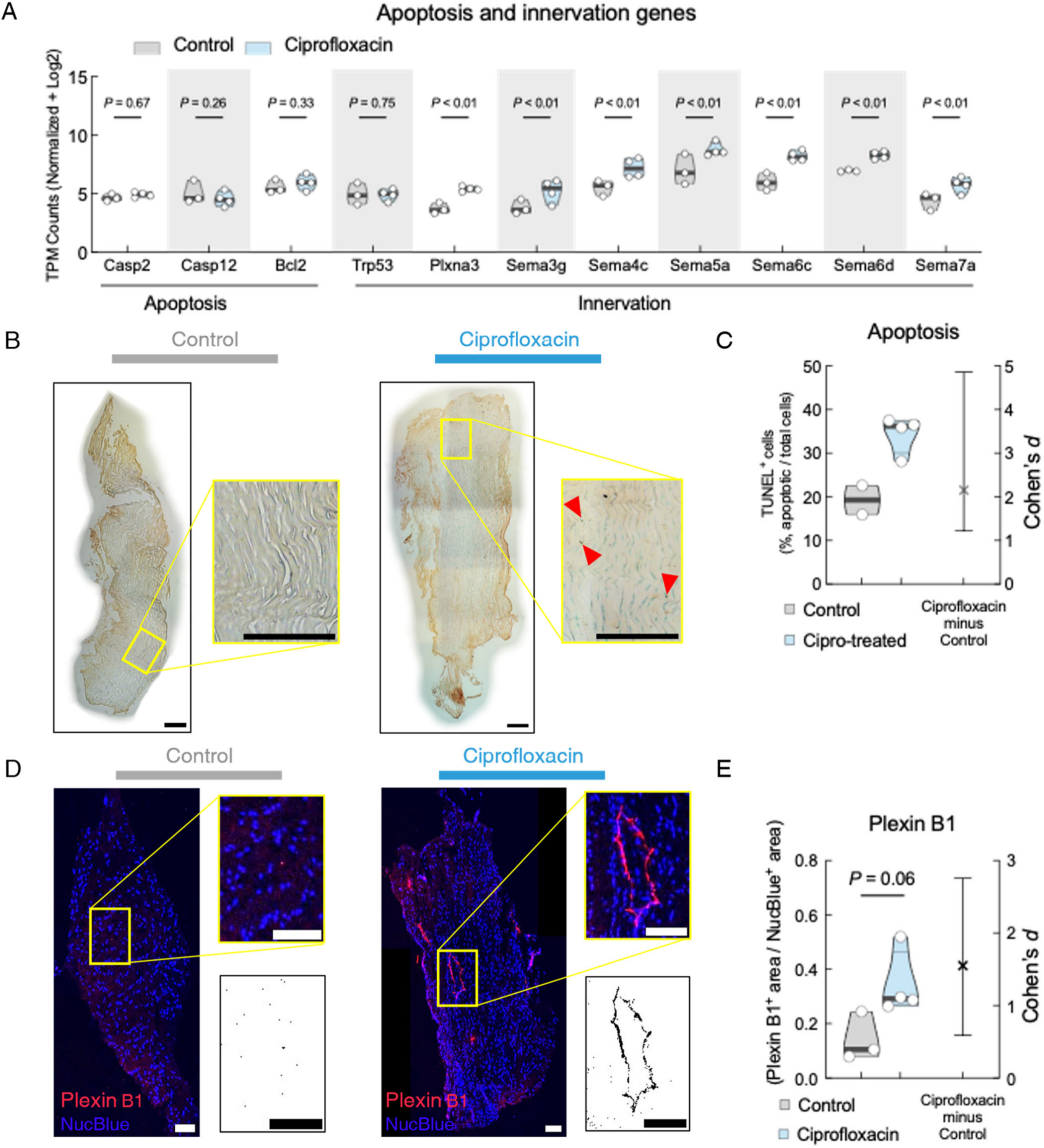
Systemic ciprofloxacin treatment upregulates apoptotic and innervation genes in tail tendons and induces apoptosis and nerve ingrowth in patellar tendons. (A) Gene analysis from RNA sequencing data, clustered by apoptotic (left) and innervation (right) genes. (B) Representative images of TUNEL staining of control (left) and ciprofloxacin‐treated (right) patellar tendon with magnified region of interest. Red triangles highlight specific examples of TUNEL+ cells based on the positive control (Supporting Figure 5). (C) Quantification of TUNEL^+^ cells normalized to total number of cells. (D) Representative images of Plexin B1 immunohistochemistry counterstained with DAPI from control (left) and ciprofloxacin‐treated (right) patellar tendon with magnified region of interest. Immediately below the magnified image is the inverted LUT of the Plexin B1 channel to further highlight differences. (E) Quantification of Plexin B1^+^ stained area normalized to NucBlue^+^ area. All scale bars are 200 μm. Violin plots show the distribution of the data with the median, first and third quartiles highlighted. Each dot represents an average from 8−20 histological sections, *n* = 2–4 mice. Analysis done with unpaired *t*‐test. *p*‐values and Cohen's d effect size are displayed.

Our transcriptome analyses on TTFs suggested the involvement of the Semaphorin‐Plexin signaling axis, which is implicated in the induction of apoptosis and nerve ingrowth. We therefore examined genes specifically related to apoptosis and nerve ingrowth, which showed an increase in the transcripts of *Plexina3* gene in TTF of the ciprofloxacin‐treated group (Figure 4A). We confirmed apoptosis in the load‐bearing PT using a TUNEL assay (Figure 4B). We found a higher percentage of apoptotic cells in the ciprofloxacin‐treated group PT (Cohen's *d* = 2.15, 95% CI [1.22, 4.86]) (Figure 4C), orthogonally confirming the RNAseq results.

Semaphorins are involved in nervous system development and axon growth and guidance [28]. Given that many genes of the Semaphorin family were upregulated in the ciprofloxacin‐treated group TTFs, we confirmed the relevance of this signaling axis by immunofluorescent staining for Plexin B1 (*Plxnb1*) on load‐bearing PTs (Figure 4D). We found a higher amount of *Plxnb1* positively stained area in the ciprofloxacin‐treated PT than in the controls (Figure 4E, *p* = 0.06, Cohen's *d* = 1.55, 95% CI [0.59, 2.76]), suggesting that *Plxnb1* and the Semaphorin family potentially play central roles in regulating the ciprofloxacin‐induced phenotype in mice.

## 4 Discussion

Overall, we observed that treating juvenile mice orally with ciprofloxacin induces structural and biological changes to their tendons potentially reminiscent of those associated with human tendinopathy. Fluoroquinolones such as ciprofloxacin are often associated with adverse tendon side effects including tendinopathy and/or tendon rupture, and this risk is less pronounced in adolescents [8]. Fluoroquinolone‐induced tendon disorders are often reversible [29]; however, this side effect was significant enough for the European Medicines Agency (EMA) to issue a recommendation to restrict fluoroquinolone use to very severe infections, and not in elderly patients or in those with musculoskeletal disorders [30].

While adverse effects of fluoroquinolones on tendons have been reported, to our knowledge there are few comprehensive in vivo studies on structural and mechanical phenotypes resulting from ciprofloxacin use on multiple tendon types. ECM degradation and MMP accumulation have been implicated as a possible cause for fluoroquinolone‐induced tendon disorders [9]. Our data show that tendons from ciprofloxacin‐treated mice have downregulated anabolic ECM genes (*Fmod*, *Comp*, *Bgn*) and upregulated catabolic ECM genes (*Adamts5*, *Adamts20*, *Mmp28)* compared to control mice, indicating turnover of the proteoglycan rich tendon ECM. An increase in these specific catabolic genes has been described in human tendinopathy [31], although not specifically in fluoroquinolone‐induced tendinopathy. CIBERSORTx analysis also predicted a potential decrease in tendon fibroblast populations in TTFs of ciprofloxacin‐treated mice. Because these populations are annotated based on *Col1a1* and *Scx* transcripts [24], we performed targeted RT‐qPCR in the PT for these genes. While possible trends emerge, there are no significant differences, potentially due to bulk tissue homogenization for RNA isolation which potentially masks small differences in transcripts.

Because most literature regarding fluoroquinolone‐induced tendinopathy describes case studies of human patients [29] or retrospective cohort studies of human patients [8, 10], we compared our phenotype to that of human tendinopathy. Tendinopathy induced by fluoroquinolones or not is characterized by a disorganized ECM [9, 25]. H&E staining of PTs from ciprofloxacin‐treated mice showed more crimped collagen fibers, characteristic of disorganized tendon structure. Human tendinopathy is also characterized by an increase in proteoglycans and GAG content [9, 25, 31]. Alcian blue staining showed an increase in proteoglycan content in the ciprofloxacin‐treated group when compared to the control group. However, a DMMB assay for sulphated GAG content normalized to DNA (a proxy of cellularity) measured no change between the groups. This null result could be in part due to DMMB dye interactions with proteins, nucleic acids, and other sulfated compounds [32] since the entire tendon was digested, including matrix components that may interfere with the analysis. Additionally, proteoglycan gene expression (*Bgn*, *Fmod*, *Dcn*) was decreased in the ciprofloxacin‐treated group; taken together with the Alcian blue observations potentially indicating that gene expression is down while protein expression is upregulated. Results from human studies of ciprofloxacin treatment indicate that glycosaminoglycan activity might decrease in the Achilles tendon after ciprofloxacin treatment [33].

Human tendinopathy is often debilitating, manifesting with pain and functional deficits [26]. Based on the structural differences we observed in the tendons of the two groups, we reasoned that there would also be functional differences. Mechanical analysis of PTs showed decreased elastic modulus and failure stress in ciprofloxacin‐treated mice when compared to controls, although not statistically significant due to limited sample size. Cohen's *d* analysis of these mechanical properties showed a moderate to large effect size, indicating practical functional consequences for altered tendon mechanics after ciprofloxacin treatment. Human tendinopathy is oftentimes characterized by an increase in CSA of the affected tendon [25, 26]. This was not measurable in the PTs we analyzed, observing similar CSAs in both groups.

Many studies have characterized biological changes of human tendinopathy. One hallmark change is cell rounding and apoptosis [25]. RNA sequencing of TTFs identified upregulated genes involved in apoptosis in the ciprofloxacin‐treated mice compared to control mice. H&E staining of PTs from ciprofloxacin‐treated mice showed significantly less elongated cells, while a TUNEL assay showed significantly more apoptotic cells. Another hallmark of human tendinopathy is pain and tenderness at the tendon site [9, 25, 26, 29]. RNA sequencing of TTFs from ciprofloxacin‐treated mice showed enrichment of genes implicating neuronal development and axon growth. Cellular deconvolution analysis also predicted a higher fraction of nerve cells in the ciprofloxacin‐treated group. Neuronal development was verified in the PT using Plxnb1 immunofluorescent staining. While Plxnb1 is not neuron‐specific, it does play a role in axon guidance; therefore, our results possibly suggest that ciprofloxacin treatment in mice may cause tendon pain or peripheral neuropathy. This observed neural activation in ciprofloxacin‐treated mice through Plexin‐b1 signaling could provide insight into the poorly understood mechanisms underlying tendon pain in fluoroquinolone‐induced tendinopathy.

Literature has reported the effect of ciprofloxacin treatment on tendons, mostly using *ex vivo* rodent models [34, 35, 36, 37]. However, these studies generally examined structural or mechanical changes in a specific tendon. Our study adopted a more comprehensive approach by evaluating both structure and function, using transcriptomic data to frame these tissue adaptations in terms of underlying biological processes. This effort revealed that ciprofloxacin treatment in mice yields a structural and biological phenotype in both positional tendons (TTFs) and load‐bearing tendons (PTs), resembling that reported in the tendinopathic disease state in humans.

This study has limitations. Tissues used for this study were repurposed from a separate study examining ciprofloxacin‐induced alterations in the aortic wall. While a clear picture emerges and conclusions are well‐supported by the data, analyses are underpowered with wide confidence intervals due to limited sample size and tissue availability. Power analysis calculations suggest a minimum sample size of 4 for histological and 15 for mechanical analyses to observe a 20% difference between the groups. Additionally, the young age of the mice and the supraphysiological administered dose of ciprofloxacin limit the direct relevance of our findings to ciprofloxacin‐induced tendinopathy in adult humans. Because the tendons in the present study were still developing, it is possible that ciprofloxacin disrupted maturation processes rather than inducing pathology analogous to adult tendinopathy. Moreover, humans typically receive intermittent bolus doses (e.g., 500–750 mg every 12 h), producing sharp plasma peaks of ~2.6–3.5 mg/L. In contrast, our mice received ciprofloxacin ad libitum in drinking water, yielding a sustained exposure profile. Based on published mouse pharmacokinetic data [38], we estimate that this regimen produced average plasma concentrations of ~2–7 mg/L with night‐time peaks of ~3–10 mg/L. These predicted concentrations overlap with or moderately exceed human Cmax, but with a flattened, sustained time course rather than a sharp bolus. Water consumption was monitored across cages, but due to the nature of the regimen, the delivered systemic dose can only be approximated. Thus, while our results demonstrate that systemic ciprofloxacin exposure in juveniles can alter tendon structure and biology, confirmation in adult mice under clinically relevant dosing schedules, with direct pharmacokinetic measurement, will be essential for translation. Furthermore, this study relies on TTFs subjected to a freeze‐thaw cycle for RNA sequencing due to tissue availability, which diminished RNA quality but nonetheless provided good transcriptional coverage. PTs have low RNA yield, requiring us to pool tissues to attain adequate RNA to permit bulk sequencing. Since the tissues were repurposed from another study and limited, RNA sequencing on PTs was impossible; therefore we used TTFs. While intrinsic differences between load‐bearing and positional tendons are acknowledged, prior studies in our group and others [39, 40, 41, 42] have shown that tail tendons are transcriptomically representative of load‐bearing tendons under systemic interventions. In this study, specific identified changes in the TTF transcriptome were verified in the PT using histology and targeted RT‐qPCR. This triangulation across RNA‐seq (TTF), histology (PT), and targeted RT‐qPCR (PT) supports the interpretation that systemic ciprofloxacin exposure induces broadly similar changes across tendon types. Nonetheless, we recognize that intrinsic differences between positional and load‐bearing tendons remain a limitation, and future studies should prioritize sequencing of Achilles or patellar tendons to maximize translational relevance, and provide more confidence in the predicted cellular distribution based on CIBERSORTx analysis.

We thus propose that oral administration of ciprofloxacin in mice offers potential as a clinically relevant model for studying mechanisms of fluoroquinolone‐induced tendinopathy. Using a combination of tail and patellar tendons, we achieved low variability results across a multi‐modal analysis sufficient to reveal a previously unrecognized neural activation component. The observed upregulation of semaphorin‐plexin signaling pathways and increased Plxnb1^+^ nerve cells provides potential mechanistic insight into the clinical phenomenon of fluoroquinolone‐associated tendon nociception. Furthermore, the observed onset of aberrant tissue remodeling in ciprofloxacin‐treated mice, combined with apoptotic signatures and cellular depletion of tendon fibroblasts, plausibly explains the propensity for tendon rupture in ciprofloxacin‐treated humans. Due to the significant limitations of this study mainly stemming from the repurpose of tissues from a separate study, cautious interpretation of this data is necessary. However, a clear phenotype in both positional and load‐bearing mouse tendons emerges that is reminiscent of human disease. The transcriptomic and structural changes observed in this study could inform the design of future studies to probe deeper into the black box warning of fluoroquinolone use, and potentially proactively mitigate side effects, allowing for more indiscriminate use of the antibiotic without fear of severe adverse effects on the patient.

## Author Contributions

N.A.C designed, performed, and analyzed mechanical testing experiments, performed histology, performed the RNA extraction and wrote the manuscript. Amro A. Hussien performed RNA sequencing analysis and created Figure 1. Gabor Matyas and Nicolo Dubacher designed the animal experiment. All authors have read, revised and approved the final version.

## Conflicts of Interest

The authors declare no conflicts of interest.

## Supporting information


**Supporting Figure 1:** Cell fraction of various cell types. **Supporting Figure 2:** Overrepresentation analysis of biological processes from mouse tail tendon fascicle transcriptome. **Supporting Figure 3:** Representative images of picrosirius red staining under brightfield microscopy. **Supporting Figure 4:** Mechanical testing setup verification. **Supporting Figure 5:** Positive and negative controls from the TUNEL assay.

## Data Availability

Data available upon request.
