# Supplementary material for: Ciprofloxacin Treatment in Juvenile Mice Involves Neuronal Activation and Mimics Physical Features of Human Disease
Source: J Orthop Res. 2025 Oct 28;44(2):e70095. doi: 10.1002/jor.70095 (PMC12865670; doi:10.1002/jor.70095)
Supplement: Supplementary file 1 — Supporting Figure 1: Cell fraction of various cell types. Supporting Figure 2: Overrepresentation analysis of biological processes from mouse tail tendon fascicle transcriptome. Supporting Figure 3: Representative images of picrosirius red staining under brightfield microscopy. Supporting Figure 4: Mechanical testing setup verification. Supporting Figure 5: Positive and negative controls from the TUNEL assay. [file JOR-44-na-s001.pdf]

Supplementary Information: Ciprofloxacin treatment in juvenile mice involves neuronal activation and mimics physical features of human disease

Supplementary Methods

RNA sequencing

Briefly, RNA integrity was measured with RNA Kit on Agilent 5300 or 5600 Fragment Analyzer (Agilent Technologies, Palo Alto, CA, USA). Total RNA was normalized using ERCC RNA Spike-In Mix kit (4456740, ThermoFisher Scientific) following manufacturer's protocol. Ribosomal RNA (rNA) was depleted using NEBNext rRNA Depletion kit (Mouse). RNA sequencing libraries were prepared with NEBNext Ultra II Directional RNA Library Prep kit for Illumina (NEB, Ipswich, MA, USA): enriched RNAs were fragmented, first and second strand cDNA were synthesized, cDNA fragments were end-repaired and adenylated at 3' ends, universal adapter was ligated to cDNA fragments, and limited cycle PCR was performed for index addition and library enrichment. Validation of sequencing libraries was performed using NGS Kit on the Agilent 5300 or 5600 Fragment Analyzer (Agilent Technologies, Palo Alto, CA, USA). Sequencing libraries were multiplexed, loaded on the flow cell of an Illumina NovaSeq 6000 instrument (or similar), and sequenced using a 2x150 Pair-End (PE) configuration v1.5 to a sequencing depth of at least 20 million reads. NovaSeq Control Software v1.7 on the NovaSeq instrument was used to perform image analysis and base calling. This generated raw sequence data (.bcl files), which were then converted into fastq files and de-multiplexed using the Illumina bcl2fastq program v2.20. One mismatch was used for index sequence identification.

Data preprocessing and bioinformatics analysis

The R package ezRun was used to process raw sequencing data via the SUSHI framework of the Functional Genomics Center Zurich (ETH Zurich and the University of Zurich) (1). Quality of the raw data was analyzed with MultiQC v1.12 (2). STAR aligner software was used to map the raw .fastq files to the Mus musculus genome reference (GENCODE GRCm39 – Release_M31-2023-01-30) (3). The featureCounts function was used with a correct step for GC bias and gene length counts to calculate the read counts per gene. The packages EdgeR v1.36.0, Enrichr v3.18.0, and clusterProfiler v3.18.0 were used to perform differential expression analysis, pathway enrichment analysis, and gene set overrepresentation analysis, respectively (4-6). Cellular deconvolution was performed using the CIBERSORTx tool and the tendon cell-type signature matrix developed by De Micheli, et al. (7-9). The VolcaNoseR package was used to create volcano plots (10).

1. Hatakeyama M, Opitz L, Russo G, et al. 2016. SUSHI: an exquisite recipe for fully documented, reproducible and reusable NGS data analysis. BMC Bioinformatics 17: 1-9.
2. Ewels P, Magnusson M, Lundin S, Käller M. 2016. MultiQC: summarize analysis results for multiple tools and samples in a single report. Bioinform 32(19): 3047-3048.
3. Dobin A, Davis CA, Schlesinger F, et al. 2012. STAR: ultrafast universal RNA-seq aligner. Bioinform 29(1): 15-21.
4. Chen EY, Tan CM, Kou Y, et al. 2013. Enrichr: interactive and collaborative HTML5 gene list enrichment analysis tool. BMC Bioinformatics 14: 1-14.
5. Robinson MD, McCarthy DJ, Smyth GK. 2009. edgeR: a Bioconductor package for differential expression analysis of digital gene expression data. Bioinform 26(1): 139-140.
6. Yu G, Wang LG, Han Y, He QY. 2012. clusterProfiler: an R package for comparing biological themes among gene clusters. Omics 16(5): 284-287.
7. De Micheli AJ, Swanson JB, Disser NP, et al. 2020. Single-cell transcriptomic analysis identifies extensive heterogeneity in the cellular composition of mouse Achilles tendons. Am J Physiol Cell Physiol 319(5): C885-C894.
8. Newman AM, Steen CB, Liu CL, et al. 2019. Determining cell type abundance and expression from bulk tissues with digital cytometry. Nat Biotechnol 37(7): 773-782.
9. Steen CB, Liu CL, Alizadeh AA, Newman AM. 2020. Profiling cell type abundance and expression in bulk tissues with CIBERSORTx. Methods Mol Biol 135-157.
10. Goedhart J, Luijsterburg MS. 2020. VolcaNoseR is a web app for creating, exploring, labeling and sharing volcano plots. Sci Rep 10: 20560.

Immunohistochemistry antibodies

Primary Ab	Manufacturer	Concentration	Secondary Ab	Manufacturer	Concentration
Plexin B1 polyclonal antibody	Thermo Fisher Scientific BS-2693R	1:200	Goat anti-rabbit IgG (H+L) secondary antibody, Alexa Fluor 647	Thermo Fisher Scientific A21244	1:250

Custom-designed jig fabrication, sensitivity testing and analysis

The jig was fabricated and designed to fit in a universal testing machine (Zwick Z010 TN). The sensitivity of the jig was tested by isolating and inserting a patellar tendon with attached tibia and patella. The tendon was inserted, immersed in 1% PBS, stretched to a preload of 0.5 N, preconditioned 25 cycles to 1% strain, allowed a 30 s rest period and then stretched to 8% strain at a speed of 5.0% L_0/s. The tendon was returned to the starting length and PBS was removed. The tendon was injected with collagenase (Col A, Roche Diagnostics, 10103578001, 0.228 U/mg lyo.) dissolved in DPBS with Ca^{2+} (ThermoFisher Scientific, 14040117) at a concentration of 12 mg/ml. After allowing the injection to disperse for 10 minutes, the tendon was immersed again in PBS and incubated at RT for 1 h. The stretching and injection protocols were repeated every hour for a total of 5 hours. Force and position data were recorded. Cross-sectional area was used to calculate stress. Elastic modulus was calculated as the linear region of the stress-strain curves, failure stress as maximum stress and failure strain as the corresponding strain value.

Supplementary Figures

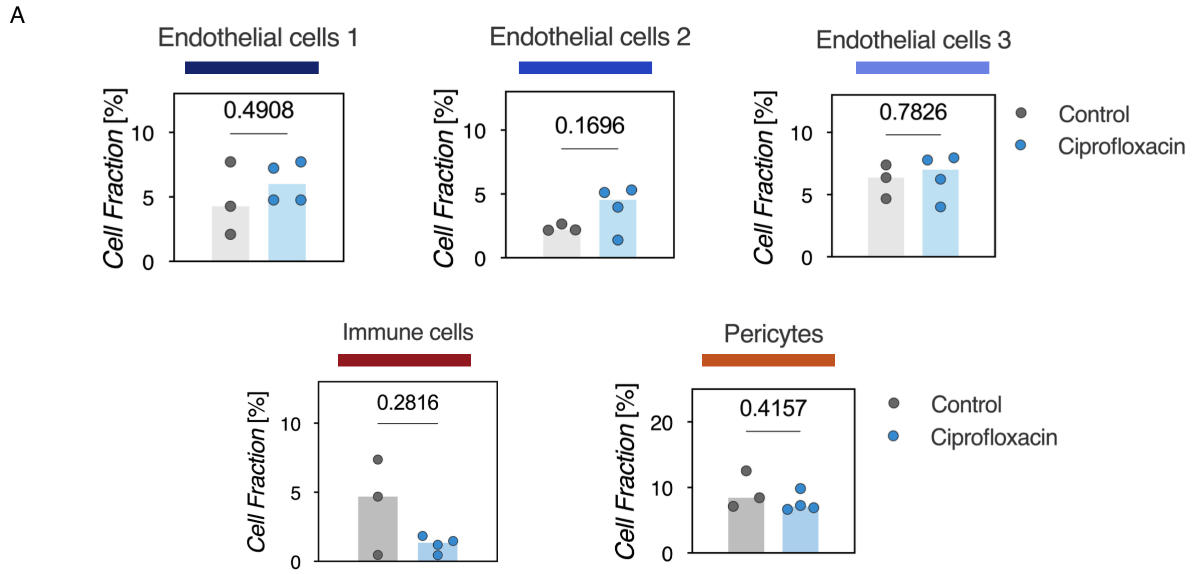

Supplementary Fig. 1. Cell fraction of various cell types.

Each point represents one animal, n = 3 for the control group and n = 4 for the ciprofloxacin-treated group.

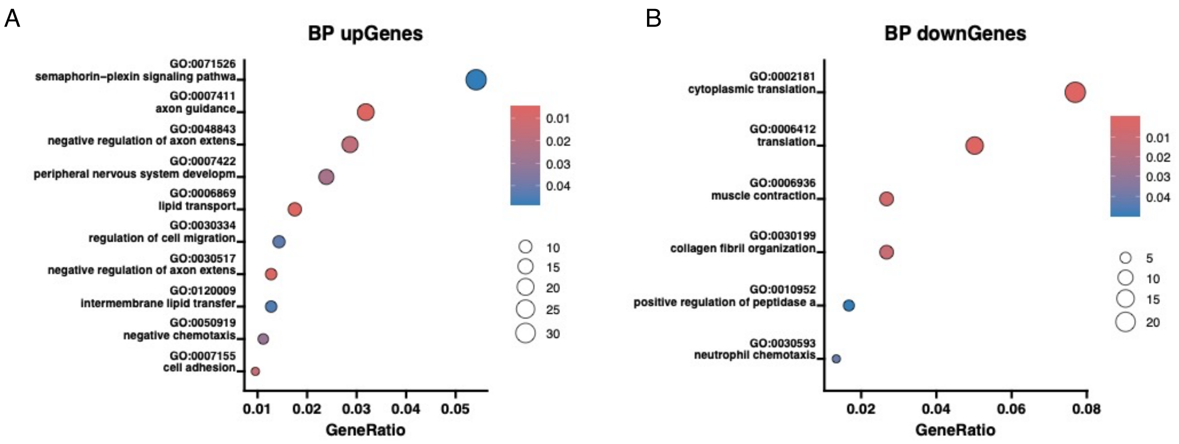

Supplementary Fig. 2. Overrepresentation analysis of biological processes from mouse tail tendon fascicle transcriptome. GO terms of (A) upregulated DEGs and (B) downregulated DEGs. Size of circle for each GO term represents counts for each GO term, and color indicates significance based on the included color scale.

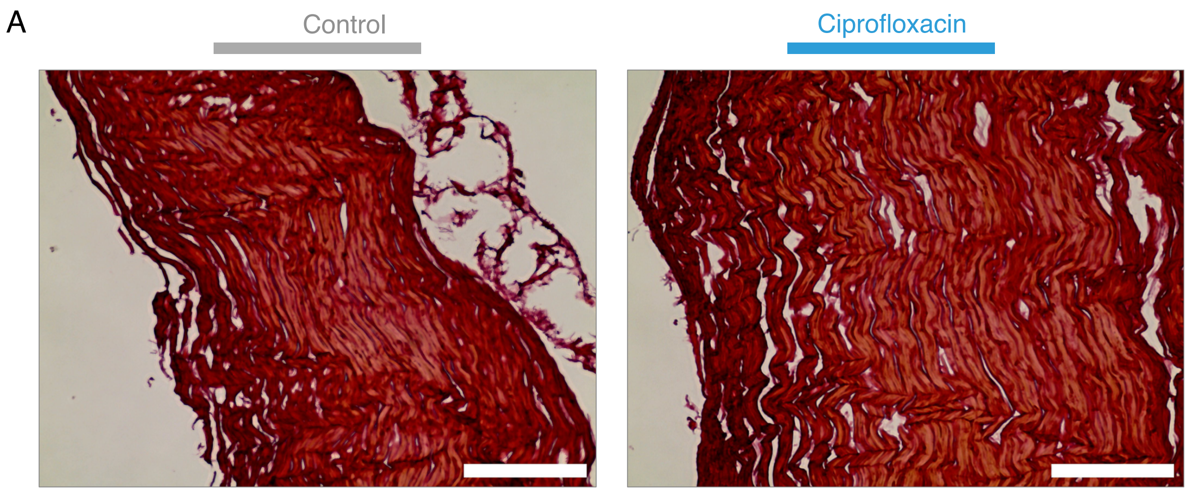

Supplementary Fig. 3. Representative images of picrosirius red staining under brightfield microscopy.

(A) Left image is from control mouse, right image is from ciprofloxacin-treated mouse. Scale bars are 200 μm.

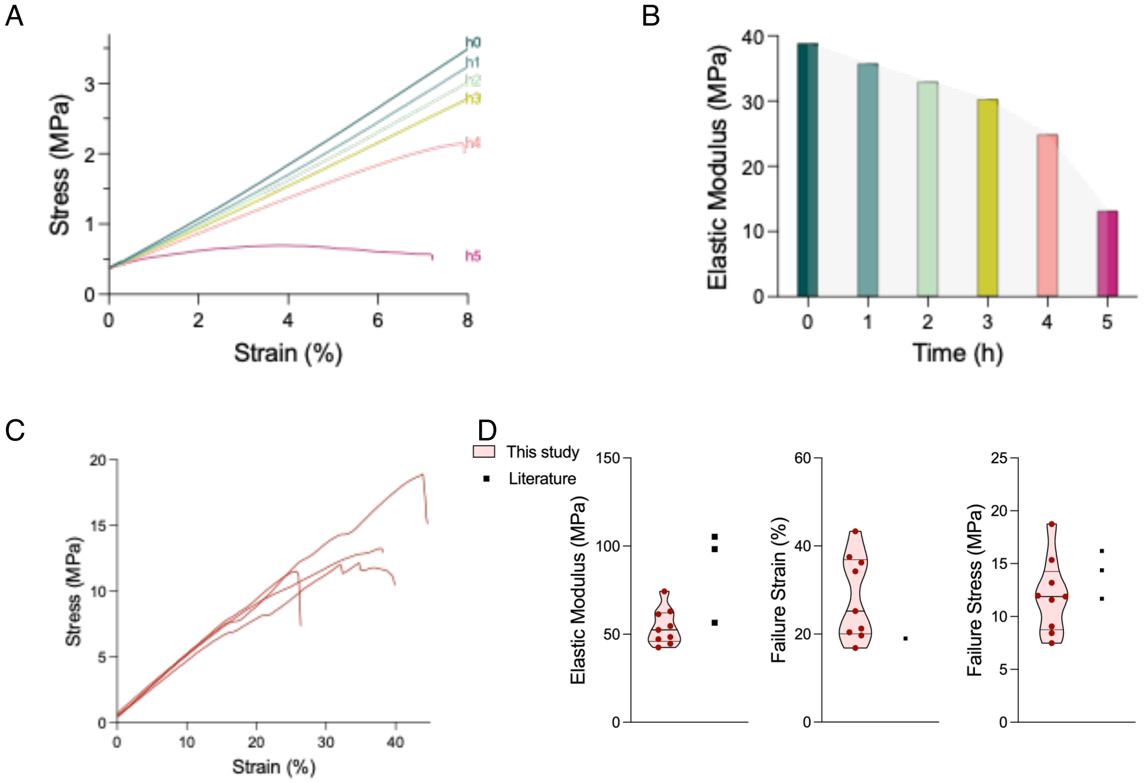

Supplementary Fig. 4. Mechanical testing setup verification.

(A) Stress-strain curves of the elastic region of the tendon (0-8%) after each hour of incubation with collagenase. **(B)** Elastic modulus values calculated from the stress-strain curves for each hour of incubation with collagenase. **(C)** Stress-strain curves of a single patellar tendon from four different mice of the same age and sex. **(D)** Mechanical properties calculated from stress-strain curves of various tests compared to literature values [15]– [17]. Violin plots show the distribution of the data with the median, first and third quartiles highlighted. Each point represents one animal, i.e., the average of the left and right patellar tendons from a single animal, n = 9. Literature data is taken from publications as median, SD and n; each point represents one mean value from one publication.

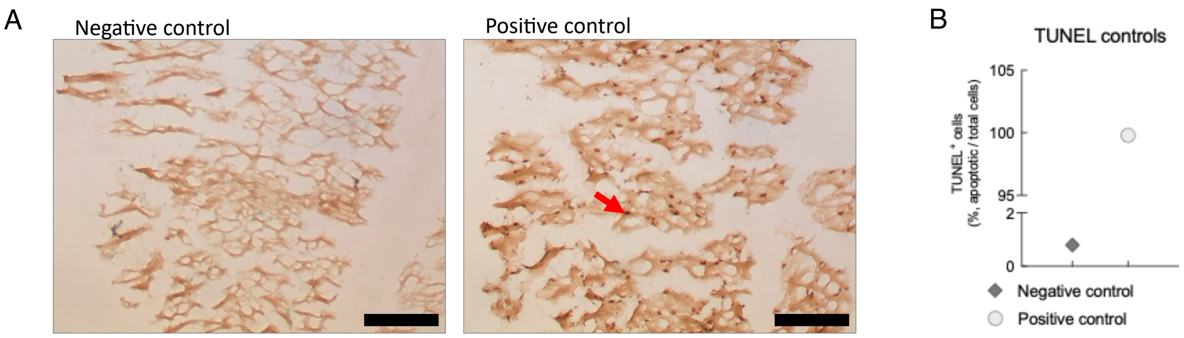

Supplementary Fig. 5. Positive and negative controls from the TUNEL assay.

(A) Representative images of controls. Red arrow points to an example of a dark-stained cell, which staining indicates is an apoptotic cell. Scale bars 200 µm. **(B)** Quantification of dark regions in positive and negative controls. Each point represents 3 samples averaged from n = 1 mouse.
